# Chitosan Enhances Intestinal Health in Cats by Altering the Composition of Gut Microbiota and Metabolites

**DOI:** 10.3390/metabo13040529

**Published:** 2023-04-06

**Authors:** Ruixia Mo, Mingrui Zhang, Haotian Wang, Tianyi Liu, Pan Liu, Yi Wu

**Affiliations:** State Key Laboratory of Animal Nutrition, College of Animal Science and Technology, China Agricultural University, Beijing 100193, China

**Keywords:** cat, chitosan, intestinal health, gut microbiota, SCFAs

## Abstract

The interaction between gut microbiota and the health of the host has gained increasing attention. Chitosan is a natural alkaline polysaccharide with a wide range of beneficial effects. However, rare studies have been observed on the effects of dietary chitosan supplementation on intestinal health in cats. A total of 30 cats with mild diarrhea were divided into three groups, receiving a basic diet with 0 (CON), 500 (L-CS) or 2000 (H-CS) mg/kg chitosan. Samples of blood and feces were collected and analyzed for serology and gut microbiota composition. The results demonstrated that chitosan alleviated symptoms of diarrhea, with enhanced antioxidant capability and decreased inflammatory biomarker levels in serum. Chitosan reshaped the composition of gut microbiota in cats that the beneficial bacteria *Allobaculum* was significantly increased in the H-CS group. Acetate and butyrate contents in feces were significantly higher in the H-CS group in comparison to the CON group (*p* < 0.05). In conclusion, the addition of dietary chitosan in cats enhanced intestinal health by modulating their intestinal microbes and improved microbiota-derived SCFA production. Our results provided insights into the role of chitosan in the gut microbiota of felines.

## 1. Introduction

The intestine of mammals is a vital organ for digestion and absorption, while also being the largest immune organ [1], thus illustrating an intricate association between intestinal health and host health status [2]. In the intestine, the most abundant and diverse microbial systems coexist, including interacting bacteria, fungi, and viruses coexisting in the intestine [3]. Following the rapid development of microbial analysis techniques, such as 16S rRNA analysis, the gut microbiota has been a critical frontier in understanding animal intestinal homeostasis and disease progression [4].

Nutritional interventions in the diet play a critical role in altering the composition and function of the host’s gut microbiota [4,5]. The structure of the gut microbiota and its metabolites in cats, which are carnivores by nature, is different from that of omnivores and herbivores [6,7]. Despite the fact that cats have a short and less functional colon, fermenting microbes are abundant in their posterior intestine [8]. Moreover, numerous studies have reported that complex bacterial communities experience significant alterations during intestinal diseases in cats [9]. Consequently, the gut microbiota and its metabolites caused by diets could affect the intestinal health of felines [10].

Therefore, it is important to investigate and understand the relationship between gut microbiota and feline diets. Dietary polysaccharides are carbohydrates synthesized by the polymerization of more than ten monosaccharides. Complex dietary polysaccharides could be metabolized into rich short-chain fatty acids (SCFAs) by intestinal microbes capable of passing through an enzyme system that is not available in mammals [11]. Acetate, propionate, and butyrate were the most abundant SCFAs [12]. SCFAs possess many beneficial effects, including providing energy to the intestine, maintaining intestinal pH, stimulating the secretion of gastrointestinal hormones, and protecting the functioning of the intestinal barrier [11].

Chitosan is a dietary polysaccharide derived from the deacetylation or partial deacetylation of chitin in arthropod and mollusk cell membranes [13]. Chitosan has many physiological properties, including antibacterial, anti-inflammatory, and immune-regulatory properties [14,15]. In addition, chitosan has been found to affect the microbial diversity in the intestine of mice, especially by lowering the ratio of Firmicutes/Bacteroidetes and decreasing Proteobacteria [16]. Concomitantly, previous research has suggested that the supplementation of dietary chitosan has decreased the abundance of pathogenic bacteria, such as *Escherichia coli*, *Shigella* spp., and *Desulfovibrio* spp. [17]. Meanwhile, in a study on rats, chitosan has been found to increase the serum concentrations of propionate and butyrate derived from gut microbiota [18]. However, research on the effects of dietary chitosan on the intestinal health of felines is scarce.

Thus, the aim of this study was to examine the effects of chitosan on immunity, intestinal health, the composition of the gut microbiota, and fecal SCFAs, and illustrate the potential dietary administration to improve the intestinal health of felines.

## 2. Materials and Methods

### 2.1. Animal Ethics Statement

The experimental protocol and animal care were approved by the CAU Animal Care and Use Committee (AW60203202-1-1). All feeding practices were performed strictly following the National Research Council’s (NRC) Guide.

### 2.2. Animals and Experimental Treatments

At the beginning of the research, a medical examination was performed on a colony of cats, which included appetite, mental status, parasites, and body condition. Thirty adult British shorthair cats (half male and half female) with an average age of 2.5 years and an average body weight (BW) of 8.33 ± 0.53 kg that had been spayed or neutered were included in the study. These cats experienced mild diarrhea with fecal scores ranging between 4 and 6, which is depicted in Appendix A Table A1. No chronic systemic or immune-mediated diseases were observed in these cats, as indicated by the absence of parasites in feces, urine, and blood. The cats had not received any antibiotics for three months before the experiment and were on the same basic diet for one month prior to the experiment.

Thirty cats were randomly assigned to three treatments with 10 replicates per group while ensuring that five males and five females were included in each treatment. The cats received a basal diet with 0 (CON), 500 (L-CS) or 2000 (H-CS) mg/kg chitosan for a period of 60 days. The diet was formulated to meet the nutrient recommendation of NRC (2006) (Table 1) [19]. The chitosan (S24914) used in this study was obtained from Shanghai Yuanye Biotechnology Co., Ltd. (Shanghai, China) and the deacetylation of chitosan was 85%. The samples of diet and feces were dried at 65 °C for 48 h, then ground through a 40 mesh (425 µm) sieve. According to the previous method [20], the samples of feed were analyzed in terms of crude protein (CP), dry matter (DM), ether extract (EE), and organic matter (OM). Gross energy (GE) was evaluated using an automatic calorimeter (Parr 6400, Moline, IL, USA). The samples of feces were analyzed in terms of fecal water content (FWC). All the samples were measured in duplicate.

### 2.3. Feeding and Sample Collection

The cats were provided *ad libitum* access to water and food throughout the entire experiment. The cat was housed in a cage (1.5 m × 1.5 m × 2.0 m) individually. Humidity and temperature were maintained at 50–60% and 23–26 °C, respectively. The cats were weighed, and their feces were collected on days 0, 30, and 60 to determine FWC, while feces were scored according to the fecal condition score (Appendix A Table A1). The feed supply and feed refusals were recorded daily to calculate the average daily feed intake (ADFI). The water consumption by the cat was also recorded daily to evaluate the average daily water intake (ADWI).

On days 0 and 60, blood samples were drawn from the forelimb veins into vacutainer tubes following 12 h of fasting. All blood samples were centrifuged at 3000× *g* for 30 min at 4 °C, and serum was collected and stored at − 20 °C for further analysis. On day 60, all the feces of the cats were collected immediately following defecation. Fecal samples were thereafter immediately snap-frozen in liquid nitrogen and stored at −80 °C for further examination of the microbial composition and concentration of SCFAs.

### 2.4. Serum Parameters Measurement

The concentrations of cytokines, such as interleukin-6 (IL-6; DY2305), interleukin-10 (IL-10; DY736), interleukin-1β (IL-1β; DY1796), tumor necrosis factor–α (TNF-α; DY2586), and superoxide dismutase (SOD; DYC3419–2) in the serum were determined using assay kits from R&D Systems (Minneapolis, USA). The levels of immunoglobulin A (IgA; OKIA00194) and malondialdehyde (MDA; OKEH02548) were assessed using the enzyme-linked immunosorbent assay (ELISA) kit from Aviva Systems Biology (San Diego, CA, USA). The concentrations of diamine oxidase (DAO; E-EL-H1241c), lipopolysaccharide (LPS; E-EL-H6108), and D-lactic acid (D-LA; E-BC-K002-M) in the serum were determined using Elabscience (Wuhan, China) ELISA kits. The levels of immunoglobulin G (IgG; FGG91-K01) were assessed using the ELISA kit from Eagle Biosciences (Amherst, NH, USA). All indicators were in accordance with the instructions of the kit manufacturer.

### 2.5. Hematological Parameters

On days 0 and 60 of the trial, the blood samples were drawn from cats, and their hematological parameters were analyzed using an automated animal blood cell analyzer (Mindray BC2800, Shenzhen, China).

### 2.6. Determination of Fecal SCFAs

The contents of fecal SCFAs were determined using gas chromatography according to our previous study [21]. These SCFAs include acetate, propionate, butyrate, iso-butyrate, valerate, iso-valerate, and caproate.

### 2.7. Fecal Microbiota Analysis

The total microbial genomic DNA of the fecal samples was extracted using the Fecal Genomic DNA Extraction Kit (Tiangen Biochemical Technology Co, Beijing, China). The V3-V4 region of the 16S rRNA gene was amplified gene was amplified with primer pairs 338F (5′-ACTCCTACGGGAGGCAGCAG-3′) and 806R(5′-GGACTACHVGGGTWTCTAAT-3′), subsequently pooled into equimolar amounts, and sequenced on the Illumina MiSeq platform to generate paired-end reads of 300 base pairs (bp). The high-quality sequences were clustered into ASVs using DADA2 in QIIME 2. Representative sequences of the 16S rRNA gene were annotated taxonomically against the SILVA 138 database with a confidence threshold of 70%. The raw data was uploaded to the database of the National Centre for Biotechnology Information (NCBI) with Sequence Read Archive (SRA) accession number PRJNA933768.

### 2.8. Statistical Analysis

Data analysis was performed using IBM SPSS Statistics 25 (Chicago, IL, USA). One-way analysis of variance (ANOVA) and Tukey’s test were utilized to determine the difference among groups. *p* < 0.05 was considered statistically significant, and 0.05 ≤ *p* < 0.10 was considered a tendency. Data were expressed as the mean and pooled standard error of means (SEM). Bar plots were generated using GraphPad Prism 8 (San Diego, CA, USA).

Microbial community analysis was performed using Quantitative Insights into Microbial Ecology 2 software. Only ASVs with a minimum abundance of two reads observed in more than two samples were retained. The differences between the microbiota in the principal coordinate analysis (PCoA) plots were calculated using the Abund-Jaccard distance. Kruskal–Wallis rank sum tests were used to analyze the α and β diversity and relative abundance (RA), as well as to statistically compare differences across various datasets. PCoA based on Abund-Jaccard distance was used to calculate the community similarity across subgroups, and analysis of similarities (ANOSIM) was used to test the differences between groups.

## 3. Results

### 3.1. Physical Characteristics

The results of dietary chitosan supplementation on the physical characteristics of cats are depicted in Table 2. On day 60, the FWC and fecal scores of cats in the CON group were higher in comparison to L-CS and H-CS (*p* < 0.05). The ADFI was significantly higher in cats on the H-CS diet in comparison to the CON group (*p* < 0.05). Between the three treatments, no differences were observed in ADWI and BW during the entire experimental period (*p* ≥ 0.05).

### 3.2. Hematological Parameters

The results pertaining to the hematological parameters of cats are presented in Table 3. On day 0, there was no difference in any of the items among the three groups (*p* ≥ 0.05), except that the MCH in the L-CS group was higher in comparison to the CON group (*p* < 0.05). Meanwhile, on day 60, no significant difference was observed in hematological parameters between the three groups (*p* ≥ 0.05).

### 3.3. Fecal SCFAs

The concentrations of SCFAs in the fecal samples of cats among the three dietary treatments are depicted in Table 4. The concentrations of acetate in fecal samples decreased in the CON group in comparison to the L-CS group and the H-CS group (*p* < 0.05). The levels of butyrate and total SCFAs in the feces of the H-CS group were higher in comparison to the CON group (*p* < 0.05). No significant difference was observed between the contents of propionate, iso-butyrate, valerate, iso-valerate, and caproate in feces among the three dietary treatments (*p* ≥ 0.05).

### 3.4. Serum Anti-Oxidative Condition

**Figure 1:**
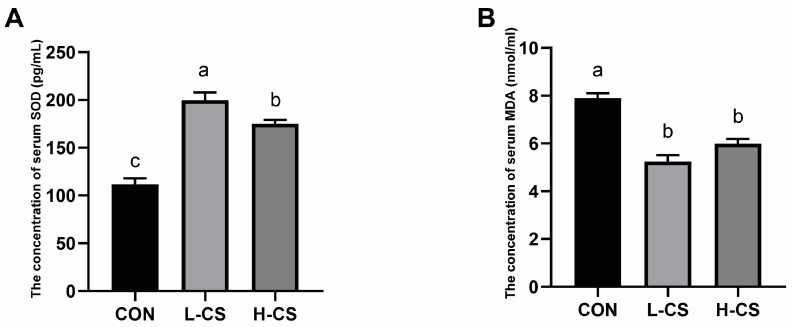
The effects of chitosan on anti-oxidative status in serum of cats: (**A**) Superoxide dismutase (SOD); (**B**) Malonaldehyde (MDA). Note: CON represents basic diet; L-CS, basic diet containing 500 mg/kg chitosan; H-CS, basic diet containing 2000 mg/kg chitosan; and SEM, standard error of means. Values are presented as means ± SEMs, *n* = 10. Different letters on the top of the column represent significant differences (*p* < 0.05).

The results for the antioxidant parameters of cats’ serum are illustrated in Figure 1. The two chitosan-supplementation groups had markedly greater serum SOD concentrations than that of the CON group (*p* < 0.05). In addition, the serum SOD concentration in the L-CS group was significantly higher than the H-CS group (Figure 1A, *p* < 0.05). Furthermore, MDA was found to be significantly reduced in the L-CS and H-CS groups in comparison to the CON group (Figure 1B, *p* < 0.05).

### 3.5. Serum Intestinal Barrier Function Parameters

**Figure 2:**
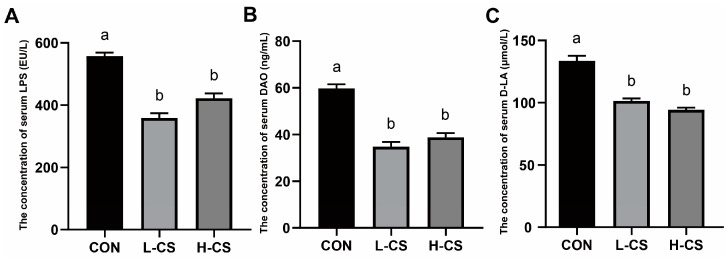
The effects of chitosan on functional parameters in the serum intestinal barrier: (**A**) Lipopolysaccharide (LPS); (**B**) Diamine oxidase (DAO); (**C**) D-lactate (D-LA). Note: CON represents basic diet; L-CS, basic diet containing 500 mg/kg chitosan; H-CS, basic diet containing 2000 mg/kg chitosan; and SEM, standard error of means. Values are presented as means ± SEMs, *n* = 10. Different letters on the top of the column represent significant differences (*p* < 0.05).

The parameters of serum intestinal barrier function in cats are illustrated in Figure 2. The serum levels of LPS (Figure 2A), DAO (Figure 2B) and D-LA (Figure 2C) were significantly lower in the L-CS and H-CS groups in comparison to the CON group (*p* < 0.05).

### 3.6. Serum Inflammatory Cytokines and Immunoglobulins

**Figure 3:**
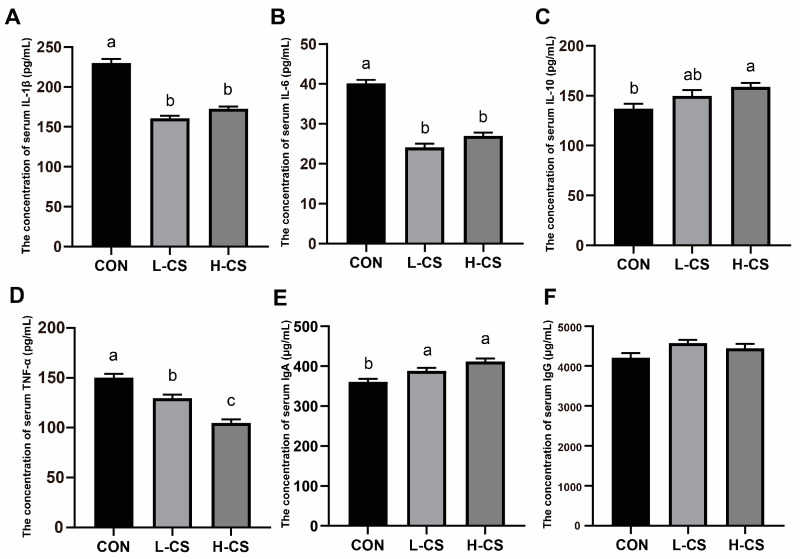
The effects of chitosan on the levels of inflammatory cytokines and immunoglobulins in the serum of cats: (**A**) Interleukin (IL)-1β; (**B**) IL-6; (**C**) IL-10; (**D**) Tumor necrosis factor (TNF)-α; (**E**) Immunoglobulin A (IgA); (**F**) Immunoglobulin G (IgG). Note: CON represents basic diet; L-CS, basic diet containing 500 mg/kg chitosan; H-CS, basic diet containing 2000 mg/kg chitosan; and SEM, standard error of means. Values are presented as means ± SEMs, *n* = 10. Different letters on the top of the column represent significant differences (*p* < 0.05).

The effects of chitosan on inflammatory cytokines and immunoglobulins in the serum of cats are illustrated in Figure 3. The levels of serum IL-1β (Figure 3A) and IL-6 (Figure 3B) in the L-CS and H-CS groups were lower than in the CON group (*p* < 0.05). Meanwhile, the level of serum IL-10 (Figure 3C) in the H-CS group was higher in comparison to the CON group (*p* < 0.05). As the content of chitosan in the diet increased, the level of TNF-α (Figure 3D) in the serum of cats gradually decreased (*p* < 0.05). The level of serum IgA (Figure 3E) was the lowest in the CON group among the three groups (*p* < 0.05). No difference was observed for IgG (Figure 3F) among the three dietary treatments (*p* < 0.05).

### 3.7. Fecal Microbiota

**Figure 4:**
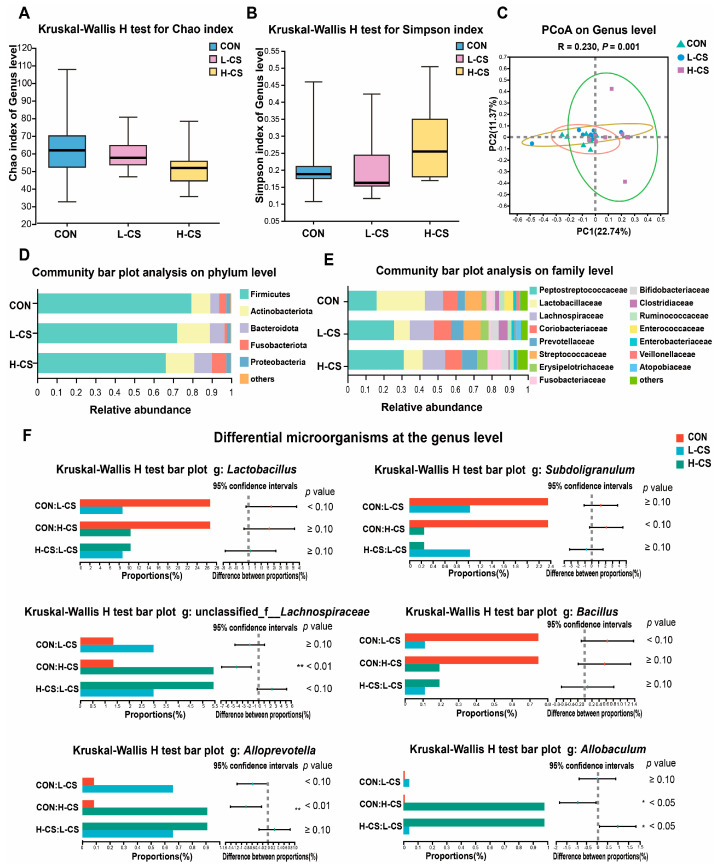
Description of the fecal microbiota composition of cats: (**A**) Comparison of alpha diversity Chao indexes in 3 dietary treatments; (**B**) Comparison of alpha diversity Simpson indexes in 3 dietary treatments; (**C**) Principal coordinate analysis (PCoA) plots of bacterial communities based on genus level; (**D**, **E**) Community bar plot analysis on phylum and family levels; (**F**) Differential microbes at the genus level in the three groups. Note: CON represents basic diet; L-CS, basic diet containing 500 mg/kg chitosan; H-CS, basic diet containing 2000 mg/kg chitosan; and SEM, standard error of means. Values are presented as means ± SEMs, *n* = 10. Significances are presented as * *p* < 0.05 and ** *p* < 0.01.

The fecal microbiota composition of cats is illustrated in Figure 4. No differences were observed among the three groups in Chao (Figure 4A) and Simpson index (Figure 4B). Furthermore, the beta diversity based on the PCoA analysis resulted in a significant separation of the fecal microbial communities among the three treatment groups (Figure 4C, *p* < 0.05). Meanwhile, the community bar plot at the phylum level revealed that Firmicutes, Actinobacteriota, Bacteroidota, Fusobacteriota, and Proteobacteria were the prevailing bacteria (Figure 4D). On the family level, the most abundant microbes were *Peptostreptococcaceae*, *Lachnospiraceae*, *Coriobacteriaceae*, *Prevotellaceae*, and *Streptococcaceae*. Interestingly, *Peptostreptococcaceae* were more prevalent in the L-CS and H-CS groups in comparison to the CON group, while *Lactobacillaceae* were the predominant bacteria in the CON group. The results of the differences in microbes at the genus level are illustrated in Figure 4F. The CON group demonstrated increased levels of *Lactobacillus* in comparison to the other two groups (*p* < 0.05). The abundance of *Bacillus* in fecal samples was significantly higher, while the abundance of *Alloprevotella* was significantly lower in the CON group in comparison to the L-CS and H-CS groups (*p* < 0.05). With increasing chitosan content in the diet, the abundance of unclassified_f_*Lachnospiraceae* in cat feces increased, whereas the abundance of *Subdoligranulum* decreased (*p* < 0.05). Meanwhile, *Allobaculum* was significantly higher in the H-CS group in comparison to the CON and L-CS groups (*p* < 0.05).

## 4. Discussion

Chitosan is a complex of β-1,4-linked d-glucosamine oligosaccharides that are deacetylated derivatives of chitin and is derived from the cell walls of crustaceans, fungi, and plants [22]. Meanwhile, chitosan is considered to be the only natural polysaccharide with alkaline and cationic characteristics conferring water absorption, antibacterial, anti-inflammatory, antioxidant, and immunological properties [23]. Various studies have assessed the efficacy of chitosan in protecting the intestinal health of animals [24,25], and this major observation is consistent with our current findings. Our results revealed that chitosan exerted beneficial effects in reducing intestinal barrier dysfunction and improving intestinal problems associated with diarrhea in cats.

Diarrhea is considered a representative symptom of impaired intestinal health [26]. In this study, 30 cats with mild diarrhea symptoms were selected, and the addition of chitosan to the diet significantly reduced the fecal water content and alleviated the symptoms in cats. This might be a result of the fact that polar groups, such as hydroxyl and amino groups contained in chitosan, are highly hygroscopic and polymeric [23]. Moreover, as a dietary polysaccharide, we found that the addition of chitosan did not compromise the dietary palatability of cats, and even the ADFI was significantly higher in cats from the H-CS group in comparison to the CON group.

The integrity of the intestinal barrier functions and the homeostasis of the intestinal microecology are important to the health of the organism [27]. The intestinal epithelial barrier is a dynamic and permeable barrier that selectively absorbs nutrients but prevents harmful substances from entering the intestinal lumen [28]. LPS is a potent pro-inflammatory molecule, and when the gut microbiota is disrupted, an excessive LPS is secreted, which could damage the intestinal mucosal barrier and release toxic substances into the bloodstream, resulting in a systemic inflammatory response [29]. D-LA is a metabolite of bacterial metabolism, and the concentration of D-LA in the serum is associated with the degree of intestinal mucosal damage [30]. DAO activity and levels are high in intestinal epithelial cells, but DAO crosses the intestinal epithelial mucosa into the serum when intestinal permeability increases [31]. Therefore, elevated serum DAO levels are a biomarker of increased intestinal permeability. Excessive accumulation of D-LA might have toxic effects on the organism because the host lacks the necessary enzymes to metabolize it [32]. These markers of intestinal barrier function, including LPS, D-LA, and DAO, pass through the intestinal mucosa to enter the circulation in significant amounts during damage to the intestinal barrier and intestinal mucosa [33]. In an earlier study with mice, it was found that the administration of chitosan reduced the levels of serum DAO and D-LA in an LPS-challenge model [34], which was consistent with our findings. On day 60, there were significant decreases in serum LPS, D-LA, and DAO concentrations in L-CS and H-CS groups in comparison to the CON group. Our results demonstrated that chitosan alleviates intestinal epithelial cell damage and reduces intestinal mucosal permeability, thereby protecting the intestinal barrier function of cats.

Impaired intestinal barrier function results in increased intestinal permeability, which exacerbates pathogen invasion of the cells [35]. At this time, the excessive immune response to pathogens increases epithelial apoptosis and decreases connexin expression, aggravating the impairment of intestinal barrier function [35]. Cytokines secreted by immune cells regulate cell function and reflect immunological health and intestinal barrier damage [36]. The increase in pro-inflammatory cytokines and decrease in anti-inflammatory cytokines in peripheral circulation is one of the typical characteristics of intestinal inflammation [37]. TNF-α is a mediator of intestinal inflammation that stimulates the secretion of IL-1β and IL-6 produced by macrophages and dendritic cells in the lamina propria [38]. In contrast, IL-10 plays role in anti-inflammatory effects [39]. IL-10 primarily inhibits nuclear factor kappa-light-chain-enhancer of activated B cell target genes in macrophages, indirectly disrupting interferon regulatory factors in dendritic and mast cells [39]. Chitosan has been demonstrated to reduce the serum concentrations of TNF-α, IL-6, and IL-1β in weaned piglet models [40]. Our results also revealed that the addition of chitosan significantly increased IL-10 and decreased TNF-α, IL-6, and IL-1β in the serum of the L-CS and H-CS groups in comparison to the CON group. IgA could be excreted from the intestine to the blood during inflammatory flare-ups and plays a significant role in suppressing inflammation [41]. In our study, chitosan significantly increased the concentration of serum IgA in cats. Therefore, these results suggested that chitosan could alleviate inflammation in cats triggered by a compromised intestinal barrier.

The release of inflammatory cytokines induces a persistent overproduction of the reactive oxygen species, which results in oxidative stress in the body [42]. The concentrations of SOD and MAD are the prevalent sensitive indicators of antioxidative and pro-oxidative systems [43]. The compromised antioxidant capacity is generally reflected by the decreased SOD and increased MDA [44]. Moreover, chitosan alleviates oxidative stress in rats by reducing MDA levels in the serum [45]. The ability of chitosan to alleviate oxidative stress was also confirmed in another study on severe acute pancreatitis, in which the level of MDA decreased, and SOD increased in the ileum and pancreas of mice following supplementation with chitosan oligosaccharides [46]. Our results in cats also confirmed the effectiveness of the antioxidant properties of chitosan, which was indicated by an increase in SOD level and a decrease in MDA level in serum following chitosan feeding.

The gut microbiota plays an essential role in keeping felines healthy and preventing diseases [7]. The inflammation of the intestine or impaired barrier is connected to dysbiosis of microbiota and the overgrowth of pathogens [1]. Previous research studies have confirmed that the composition of the gut microbiota in cats is strongly correlated with gastrointestinal health problems, such as chronic enteritis and diarrhea [9]. Similar to a previous study [7], our findings revealed that Firmicutes, Actinobacteriota, and Bacteroidota are the most prevalent phyla in the feces of cats. Moreover, following the addition of chitosan, significant differences in microbial composition and structure were observed among the three treatments. A previous study revealed that the addition of chitosan lowered the abundance of *Lactobacillus* in weaned piglets [47]. In addition, during the in vitro fermentation of chitosan by the mouse gut microbiota, *Lactobacillus* was significantly reduced following 8 h chitosan treatment [17]. These results are consistent with our finding that the supplementation of chitosan in the diet reduced the abundance of *Lactobacillus* in the feces of cats. Notably, chitosan has been reported to possess an inhibitory effect on beneficial bacteria, including *Bifidobacterium,* in contrast to traditional prebiotic polysaccharides [17,48,49]. This is probably a result of the inhibitory effect of chitosan on Gram-positive bacteria [23]. The positively charged chitosan could interact electrostatically with negatively charged alginate in the peptidoglycan of Gram-positive bacteria, resulting in cell membrane disruption and leakage of intracellular components, causing bacterial death [50]. Additionally, in this finding, *Allobaculum* was significantly enriched in the H-CS group. *Allobaculum* is an efficient glucose metabolizer and producer of lactate and butyrate, which is also believed to be involved in inflammatory processes and could have a beneficial influence on the intestinal immune responses of the host [51]. In brief, our results found that chitosan affected the composition of intestinal microorganisms in cats, but the mechanism of chitosan action needs to be further investigated.

Intestinal microbes are able to ferment indigestible carbohydrates to produce SCFAs, primarily including acetate, propionate, and butyrate [11]. The beneficial functions of SCFAs, such as reducing the production of pro-inflammatory factors and enhancing the intestinal mucosal barrier, have been extensively reported [52]. Our study revealed that the total fecal SCFAs in the H-CS group was significantly higher in comparison to that in the CON group. Butyrate provides energy to intestinal epithelial cells through fatty acid oxidation, which regulates the health of the intestine and reduces the incidence of diarrhea and enhances the immunity of the host [53]. Furthermore, acetate stimulates an immune response against pathogens and protects intestinal function by increasing IgA secretion [54]. Our study revealed significantly higher levels of fecal acetate and serum IgA in the L-CS and H-CS groups in comparison to the CON group following the addition of chitosan. Meanwhile, the level of butyrate in the feces of cats gradually increased with the addition of increased chitosan, and the level of butyrate in the feces of the H-CS group was significantly higher in comparison to the CON group. *Allobaculum*, which has been reported to be an efficient butyrate producer, is significantly elevated in the H-CS group [55]. These results revealed that dietary chitosan supplementation impacts the SCFAs production-related bacteria, which ameliorated the intestinal barrier dysfunction and intestinal health of cats.

## 5. Conclusions

In conclusion, our study demonstrated that dietary chitosan supplementation improved mild diarrhea in cats, which may be attributed to the ameliorated inflammatory and redox status as well as the increase in SCFA and beneficial bacteria. Our study provides support for the incorporation of chitosan in pet foods to protect the intestinal health of pet cats.

## Figures and Tables

**Table 1:** Ingredients and nutrient levels of basal diet.

Ingredients (As-Fed Basis)	%
Chicken meal	54.6
Fish meal	3.21
Rice	5.35
Poultry fat	9.73
Fish fat	2.15
Potato starch	18.2
Potatoes	4.85
Taurine	0.24
Chicken liver powder	0.60
Salt	0.49
Vitamin premix ^1^	0.36
Mineral premix ^2^	0.24
Total	100
**Analyzed nutrient levels (on a DM basis)**	
Dry matter, %	90.4
Total energy, MJ/kg	23.4
Crude protein, %	41.3
Ether extract, %	22.2
Ash, %	8.67

Note: ^1^ Vitamin premix provided the following per kilogram of feed: vitamin A (15,000 IU), vitamin B_1_ (30 mg), vitamin B_2_ (28 mg), vitamin B_3_ (110 mg), vitamin B_5_ (85 mg), vitamin B_6_ (12 mg), vitamin B_12_ (0.19 mg), vitamin D_3_ (15 IU), and vitamin E (75,300 IU). ^2^ Mineral premixes provided the following per kilogram of feed: Ca (CaI_2_) 20 mg, Co (CoSO_4_) 0.10 mg, Cu (CuSO_4_) 3 mg, Fe (FeSO_4_) 50 mg, I (CaI_2_) 40 mg, Mn (MnSO_4_) 18 mg, Na (Na_2_SeO_3_) 0.05 mg, Zn (ZnSO_4_) 38 mg, and Se (Na_2_SeO_3_) 260 mg.

**Table 2:** Effect of chitosan on physical characteristics of cats.

Items	CON	L-CS	H-CS	SEM	*p*-Value
**BW, kg**					
d 0	8.340	8.310	8.326	0.097	0.993
d 30	8.451	8.763	8.656	0.118	0.563
d 60	8.557	8.862	8.811	0.096	0.391
**FCW, %**					
d 0	78.12	78.51	77.51	0.750	0.878
d 30	76.87	73.33	74.37	0.642	0.064
d 60	75.81 ^a^	70.04 ^b^	71.42 ^b^	0.599	<0.01
**Fecal scores**					
d 0	5.300	5.300	5.300	0.098	1.000
d 30	5.100	4.300	4.600	0.146	0.074
d 60	4.700 ^a^	3.100 ^b^	3.700 ^b^	0.198	0.001
**ADFI, g/d**	90.62 ^b^	94.24 ^ab^	99.04 ^a^	1.427	0.048
**ADWI, mL/d**	137.2	148.7	152.0	2.924	0.092

Note: CON represents basic diet; L-CS, basic diet containing 500 mg/kg chitosan; H-CS, basic diet containing 2000 mg/kg chitosan; SEM, standard error of means; BW, body weight gain; d, day; FWC, fecal water content; ADFI, average daily feed intake; and ADWI, average daily water intake. ^a,b^ Within a row, means without a common superscript differ at *p* < 0.05. Values are presented as means and pooled SEMs, *n* = 10.

**Table 3:** Hematological parameters of cats on d 0 and 60 of the experiment.

Items	CON	L-CS	H-CS	SEM	*p*-Value
**d 0**					
WBC, 10^9^/L	9.810	9.210	10.25	0.359	0.510
Lymphocyte, 10^9^/L	3.600	3.900	3.780	0.277	0.912
Monocyte, 10^9^/L	0.500	0.480	0.410	0.034	0.550
Granulocyte, 10^9^/L	6.070	6.470	6.820	0.446	0.801
Lymphocyte, %	30.88	28.18	27.07	1.696	0.657
Monocyte, %	5.080	4.590	4.870	0.280	0.786
Granulocyte, %	51.97	49.63	49.23	2.282	0.877
RBC, 10^12^/L	7.691	7.275	6.493	0.298	0.258
Hemoglobin, g/L	121.1	118.3	120.6	3.085	0.930
Hematocrit, %	39.41	37.97	37.99	0.968	0.796
MCV, fL	45.40	46.05	45.05	0.578	0.786
MCH, pg	14.31 ^b^	15.50 ^a^	15.38 ^ab^	0.212	0.035
MCHC, g/L	318.7	328.5	331.3	3.466	0.307
RDW, %	16.02	15.51	15.71	0.182	0.532
PLT, 10^9^/L	250.6	246.0	238.8	19.80	0.972
MPV, fL	9.600	9.020	8.830	0.285	0.532
PDW	15.92	15.33	14.92	0.181	0.072
Plateletcrit, %	0.282	0.253	0.237	0.024	0.759
Eosinophil, %	2.860	3.710	3.970	0.358	0.431
**d 60**					
WBC, 10^9^/L	9.560	10.37	10.79	0.480	0.584
Lymphocyte, 10^9^/L	3.700	3.790	3.100	0.298	0.606
Monocyte, 10^9^/L	0.600	0.560	0.570	0.046	0.937
Granulocyte, 10^9^/L	6.320	6.720	6.580	0.440	0.936
Lymphocyte, %	31.70	32.48	27.57	1.498	0.368
Monocyte, %	5.330	5.090	5.220	0.250	0.931
Granulocyte, %	49.48	50.85	49.08	1.817	0.922
RBC, 10^12^/L	7.334	7.369	7.134	0.244	0.919
Hemoglobin, g/L	121.7	120.2	119.6	3.107	0.963
Hematocrit, %	38.46	37.41	38.82	1.053	0.860

Note: CON represents basic diet; L-CS, basic diet containing 500 mg/kg chitosan; H-CS, basic diet containing 2000 mg/kg chitosan; SEM, standard error of means; d, day; WBC, white blood cell; RBC, red blood cell; MCV, mean corpuscular volume; MCH, mean corpuscular hemoglobin; MCHC, mean corpuscular hemoglobin concentration; RDW, red blood cell volume distribution width; PLT, platelet count; MPV, mean platelet volume; and PDW, platelet distribution width. ^a,b^ Within a row, means without a common superscript differ at *p* < 0.05. Values are presented as means and pooled SEMs, *n* = 10.

**Table 4:** Effect of chitosan on fecal SCFAs in cats.

Items (mg/kg)	CON	L-CS	H-CS	SEM	*p*-Value
Acetate	3378 ^b^	4079 ^a^	4564 ^a^	143.0	<0.01
Propionate	2703	2906	3179	209.6	0.665
Butyrate	1885 ^b^	2298 ^ab^	2645 ^a^	100.4	<0.01
Iso-butyrate	217.6	226.1	254.1	19.04	0.729
Valerate	988.4	1037	1012	91.45	0.978
Iso-valerate	467.1	510.7	497.4	39.38	0.905
Caproate	69.81	70.34	77.97	10.72	0.945
Total SCFA	9710 ^b^	11128 ^ab^	12229 ^a^	345.8	<0.01

Note: CON represents basic diet; L-CS, basic diet containing 500 mg/kg chitosan; H-CS, basic diet containing 2000 mg/kg chitosan; SEM, standard error of means, and SCFA, short-chain fatty acid. ^a,b^ Within a row, means without a common superscript differ at *p* < 0.05. Values are presented as means and pooled SEMs, *n* = 10.

## Data Availability

For this study, data are available from the corresponding authors upon request. Data is not publicly available due to privacy.

## Appendix A

**Table A1:** Fecal condition score.

**Score**	**Standard**
1	Dry, hard, easy to pick up, difficult to defecate, and forms a ball of dung
2	Forming, not hard, segmented, and easy to pick up
3	Wet, no section, and not easy to pick up
4	Wet, columnar, and difficult to pick up
5	Wet, heaped, and hard to pick up
6	Shapeless and heaped/shoal
7	Watery diarrhea
